# On the Role of Teachers’ Professional Identity and Well-Being in Their Professional Development

**DOI:** 10.3389/fpsyg.2022.913708

**Published:** 2022-05-26

**Authors:** Qian Zhao

**Affiliations:** Faculty of Teacher Education, Pingdingshan University, Pingdingshan, China

**Keywords:** teachers’ professional identity, professional development, well-being, education, academic

## Abstract

In the former 20 years, professional identity has arisen as a different study domain in the area of language instruction and education. Educators’ professional identity frames educators’ explanation of their functions, academic changes, and alterations in syllabi, class exercises, utilization of approaches and strategies, and their connection to other problems in the academic setting. Also, well-being is one of the elements in which its influence and connection with identity are noted in various studies. However, their roles in professional development have not been examined; hence, this review of literature aimed to investigate the role of these constructs in professional development which is regarded as a noteworthy aspect of teacher quality. In a nutshell, some implications are proposed for the scholastic stakeholders.

## Introduction

Within the past few decades, practitioners, scholars, and policymakers have increasingly focused on professional development (PD) and considered the reality that educators determine new necessities and criteria for the quality teaching system (Živković, 2013). Indeed, PD refers to growing consciousness in educators about the way it works, the way they could enhance their work, their educational professionalization, and their overall education career (Beara and Okanović, 2010). As a socio-cultural phenomenon, PD refers to a website wherein language educators can deepen pedagogical content material understanding and they can reform their professional identity (PI; Borg, 2017). Undoubtedly, educators utilized PD to educate themselves, reform working activities, think about professional identities and keep passionate about education. To be professionally developed teachers, it needs to take the presence of a series of the outer and inner state of affairs, while their PI should be at the center of attention (Hanna et al., 2020). Indeed, taking the entity of a variety of outer and inner situations is needed for educators’ success and PD while focusing on educators’ PI entity (Sharma and Pandher, 2018). Educators’ PI is an important field of research and a significant subject in the area of education, mainly for the past three decades that indicates a viewpoint toward a career and one’s professional function (Wang et al., 2018; Derakhshan et al., 2020). Within the education field, PI is typically described as a group of perceptions and notions concerning how people consider themselves as educators and it refers to the fact that a person comprehends the effect and the significance of his job in the community that provides the mental basis for the influential execution of work and realizes the organizational objectives (Zhang and Wang, 2018). Teachers’ PI is considered as a working record-based group of educators’ understanding of themselves as expert actors and it includes people’s contemporary professional interests, viewpoints on education and learners’ achievement, and potentialities in the future (Sutherland et al., 2010). Developing PI can be attributed to internalizing information, competencies, viewpoints, professional values, and ethical standards that are then merged into individual personalities and manners in nursing teaching and activity (Qiu et al., 2019). Taking part in the social and cultural activities of the educator society, they achieve identification as participants of the educator society, and step by step change from an outsider to an insider in the educators’ community (Dahl, 2020). Consistent with Pillen et al. (2013), educators could employ PI as an asset for justifying themselves and academic alterations and for informing their tasks and educational activities.

On the other hand, teachers’ competence principally is determined by their well-being which is the other mental component to be taken into account while assessing PI and professional commitment which is claimed to be crucially essential to each educator and the entire educational structure (Lauermann and Konig, 2016). In agreement with the hypothesis of Positive Psychology (PP), well-being is regarded as a multi-faceted factor, whose predictors are personal, mental, and societal well-being (Wang et al., 2021). It is known to have a vital role in the quality of educators’ lives, learners’ well-being, and the steadiness of educational organizations (Wang et al., 2021). Similarly, Strauser et al. (2008) reported that well-being is a concept that greatly influences the PD cycle, and in this way, as mental well-being elevates, job-relevant stress is reduced, and the PD cycle becomes more successful. It should be mentioned that this causal relationship is true in both ways. Namely, PI influences well-being and the other way around as there is a dynamic connection between identity and well-being. Identity is scrutinized regarding the interplay of three cycles: commitment, in-depth examination, and adjustment of commitment (Crocetti et al., 2008). A teacher’s PI and their sense of competence are crucially involved in determining the commitment they have to their careers, which also links with their sense of identity and, consequently, well-being (Skinner et al., 2021). As stated by Mofrad (2016), teaching is both a matter of implementing a decontextualized set of abilities and knowledge and a dynamic cycle dependent on the educators’ particular attributes and contextual elements. As numerous contextual elements have a function in the progress of PD, it appears to be a requirement to examine the constructs that affect this process. Moreover, based on the researcher’s knowledge, there is a lack of review of literature in this domain that takes the PP issues and teachers’ PI together. Hence, investigating the PI of educators and other crucial elements, such as their well-being are prominent aspects of this review.

## Review of Literature

### Teacher Professional Development

The notion of educator PD idea has been developed and modified to encompass real execution of information and competencies in educator activities and ultimately, enhancement of the learners’ learning (Darling-Hammond et al., 2017). The educators’ PD is the formal and informal uptake of learning visions that expand and deepen educators’ professional skills, encompassing information, incentive, and self-regulating competencies (Richter et al., 2011). Similarly, Avalos (2011) emphasized that learning, assistance, and cooperation are vital factors in specifying the achievement of PD. The educators’ growth and professional learning pertain to the growth and improvement of educator knowledge resulting in the modification of their activities to enhance learners’ learning (Opfer and Pedder, 2011).

### Professional Identity

Professional identity is described as a group of understandings and notions concerning how people consider themselves as educators (Fejes and Köpsén, 2014). The educator’s PI means the educators’ picture of themselves and recognizing that they belong to the group of professional educators (Li and Qiu, 2016). An educator’s PI also includes peoples’ professional favorites, objectives, values, and ideas (Ruohotie-Lyhty and Moate, 2016). Moreover, Van Veen and Sleegers (2009) presented a more extensive image of factors regarding the educators’ PI, specifying career incentives, central tasks, self-respect ideas about education, the topic of education, and education as work. Furthermore, Professional commitment shapes an essential factor of educators’ PI. Professional commitment is described as the integration of educators’ professional favorites, central values, desires, identifications, and perspectives in their significant functions (Tao and Gao, 2017). Importantly, identity is described by peoples’ subjective mental experience, instead of several concepts of goal facts of the “real self” (Vignoles et al., 2006). People can build their social or work identities based on the role from dimensions of their career or their institute because PI is people’s image of themselves, particularly the group of ideas, values, reasons, and experiences that individuals employ to describe themselves in their career potential (Slay and Smith, 2011).

### Well-Being

Well-being is a notion that describes peoples’ individual constructive professional experience, and it includes five eudemonic dimensions, such as relational skill at work, development at work, the feeling of capability, discerned appreciation at work, and a desire for participating at work (Wang et al., 2021). Educators’ well-being is a constructive emotional situation caused through a balance among the amplitude of specific contextual factors, personal requests, and expectations considering the school (Engels et al., 2004). Studying well-being was in two mechanisms, subjective well-being and mental well-being which are, respectively, assessed by hedonic and eudemonic methods. Contrary to the hedonic viewpoint, the eudaimonic addresses human struggling that is featured as a psychosocial growth that consists of success and proper links, feeling competent and decisive, and admitting that life is important and deliberate and emphasizes seriousness along with satisfaction (Diener et al., 2010). PP aims at flourishing and alludes to well-being by employing high degrees of PERMA in life. It indicates constructive feelings, involvement with activities that take into account peoples’ strengths, enhancing positive individual rapport, figuring out the meaning by aiding a motive beyond oneself, and specifying elements of success and achievement (Seligman, 2018). By this version, well-being comes from the positivity relations in all these factors which refer to the eudemonic perspective of wellbeing (Mercer and Gregersen, 2020).

### Empirical Studies

Jue and Ha (2018) examined the professional identity, job commitment, and well-being of art therapy learners in comparison with those of psychology learners. The results of their study through correlation displayed that professional identity is consistent with job commitment and well-being. Indeed, they concluded that these three concepts are significant for the identification of art therapy graduate learners’ PD. Moreover, Stenberg (2011) tries to promote student trainers’ PD with the support of identity work that refers to replicating not only individual but also practiced skills. The findings concluded the four diverse techniques of working with identities, each of them illuminating diverse facets of and methods to identity work and it is indicated that identity work has a significant relation with PD.

## Conclusion

Since education knowledge is the core of educators’ teaching so knowing about the educators and their personalities is crucial in comprehending the procedure of education (Varghese et al., 2005) and PD. For an instance, identity is an effective element of educators’ commitment, pleasure, and incentive in changing instructional, political, and social settings wherein they act and it is an effective path to apprehend the activity and the way educators decide in the process of performing activities in the PD (Enyedy et al., 2005). As a result, educators’ PD must be formulated in close association with a quest of PI by how they characterize themselves and others. It is a development of the professional I, which develops throughout their profession and might be affected by the school, political changes, and contexts, and it is founded on individuals’ commitment, the presence to learn and instruct principles, and information on the topics they teach and how they teach them, their former experiences and professional helplessness. Furthermore, it influences their PD and assists them with managing academic alterations, bringing innovation and well-being into the class, and being creative in their teaching practice.

An educator’s PI, attained and intermediated through communications with others, is greatly included in well-being. The commitment educators have to their job also interweaves with their sense of identity and thus their well-being (Skinner et al., 2021). Moreover, educators’ well-being offers factors of fulfillment among educators; therefore, it would also be linked to how educators feel regarding the supporting factors in the school setting. With the consciousness of the knowledge and abilities, they have mastered through PD exercises, in addition to the help attained from associations (achieved through identity aspects), educators are prone to be more successful in their instructing career. Individuals with a strong PI usually take pride in their careers and attain development and self-realization through their careers. Moreover, such a strong PI is greatly connected with personal well-being in the PD (Jue and Ha, 2018). Regarding the importance of identity in PD and depending on how educators observe themselves and want to be observed by others, their teaching identities in their teaching circumstances should be developed through communication with other co-workers, pupils, supervisors, and the teaching approaches and materials that can be done through PD activities.

## Implications and Suggestions for Further Research

It can be inferred that learning PI is important since educators’ discernments of their own PI influence their PD and their capability and inclination to manage academic alteration and to apply innovations in their own instructing practice. Educators possess their peculiar reasoning of the syllabus plans and their particular strategies of instructing that are fastened to their identities; therefore, comprehending educators’ identities is required for high-standard mastering of the career in a continuous paradigm of academic change. Also because of the intricacies of identity, educator trainers in higher education organizations should pay more attention to educators’ identity deeply. By taking time for in-service educators to become aware of their own identities, and their suggestions, and offering space for individual contemplation, it might be possible to make identities more logical to unanticipated changes. In other words, a consciousness of identities might have a significant function to improve educators’ ownership of identities in their professional trajectories because numerous elements make contributions to educators’ belief of their PI which includes education, teaching, job setting, years of working experience, and so on, thus, further research should be accomplished to consider them to help shaping educators’ PI. In theoretical works, it is comprehensively acknowledged that educators’ identity is a critical aspect of their progress so investigating educators’ PI has remarkable applications for educational program reforming, class education, and learner learning. This review has feasible suggestions for those working with teachers in educator training and PD throughout their professions.

## Author Contributions

The author confirms being the sole contributor of this work and has approved it for publication.

## Conflict of Interest

The author declares that the research was conducted in the absence of any commercial or financial relationships that could be construed as a potential conflict of interest.

## Publisher’s Note

All claims expressed in this article are solely those of the authors and do not necessarily represent those of their affiliated organizations, or those of the publisher, the editors and the reviewers. Any product that may be evaluated in this article, or claim that may be made by its manufacturer, is not guaranteed or endorsed by the publisher.
